# In vitro and in vivo characterization of highly purified Human Mesothelioma derived cells

**DOI:** 10.1186/1471-2407-10-54

**Published:** 2010-02-22

**Authors:** Alice Melotti, Antonio Daga, Daniela Marubbi, Annalisa Zunino, Luciano Mutti, Giorgio Corte

**Affiliations:** 1 Department of Oncology, Biology and Genetics, University of Genova, Genova, Italy; 2 Department of Translational Oncology, National Institute for Cancer Research, Genova, Italy; 3 Department of Medicine, Local Health Unit 11, Piemonte, Italy

## Abstract

**Background:**

Malignant pleural mesothelioma is a rare disease known to be resistant to conventional therapies. A better understanding of mesothelioma biology may provide the rationale for new therapeutic strategies. In this regard, tumor cell lines development has been an important tool to study the biological properties of many tumors. However all the cell lines established so far were grown in medium containing at least 10% serum, and it has been shown that primary cell lines cultured under these conditions lose their ability to differentiate, acquire gene expression profiles that differ from that of tissue specific stem cells or the primary tumor they derive from, and in some cases are neither clonogenic nor tumorigenic. Our work was aimed to establish from fresh human pleural mesothelioma samples cell cultures maintaining tumorigenic properties.

**Methods:**

The primary cell cultures, obtained from four human pleural mesotheliomas, were expanded in vitro in a low serum proliferation-permissive medium and the expression of different markers as well as the tumorigenicity in immunodeficient mice was evaluated.

**Results:**

The established mesothelioma cell cultures are able to engraft, after pseudo orthotopic intraperitoneal transplantation, in immunodeficient mouse and maintain this ability to after serial transplantation. Our cell cultures were strongly positive for CD46, CD47, CD56 and CD63 and were also strongly positive for some markers never described before in mesothelioma cell lines, including CD55, CD90 and CD99. By real time PCR we found that our cell lines expressed high mRNA levels of typical mesothelioma markers as mesothelin (MSLN) and calretinin (CALB2), and of BMI-1, a stemness marker, and DKK1, a potent Wingless [WNT] inhibitor.

**Conclusions:**

These cell cultures may provide a valuable in vitro and in vivo model to investigate mesothelioma biology. The identification of new mesothelioma markers may be useful for diagnosis and/or prognosis of this neoplasia as well as for isolation of mesothelioma tumor initiating cells.

## Background

Malignant mesothelioma is an aggressive neoplasm arising from the surface serosal cells of the pleural, peritoneal, and pericardial cavities. Asbestos exposure has been established as the primary cause of mesothelioma; however, there is a long latency period of 30-45 years between exposure to asbestos and development of disease. Workers in the shipyard industry, insulation workers, construction workers, and asbestos miners and manufacturers seem to be at highest risk for developing the disease. Many investigators have suggested that Simian virus 40 (SV40), originating from contaminated poliovirus vaccines that were administered in USA and some European countries between 50's and 70's, might function as a cocarcinogen involved in the development of the disease. However, the relationship between SV40 and mesothelioma remains uncertain [1]. Other findings suggest at least a cooperative carcinogenic effect of SV40 and asbestos in mesothelioma development [2,3]. The male to female ratio is about 4:1 [4]. Mesothelioma is a relatively rare disease. The incidence of mesothelioma in Western Europe, and has been steadily rising over the last 40 years, and now is of about 5000 cases per year. It is expected to reach a peak approximately in 2020, and the decrease over the next 50-60 years as a result of the implementation of rules to reduce workplace exposure to asbestos. Malignant pleural Mesothelioma (MM) most commonly develops in the fifth to seventh decade of life, with a median age of 60 years at diagnosis. The most common symptoms at diagnosis are dyspnea and nonpleuritic chest pain. Several prognostic factors have been identified in MM. Poor prognostic variables include: nonepithelial histology, older age (greater than 75 years), pleural primary, chest pain at presentation, poor performance status and elevated platelet count (greater than 400,000/mcL). The median survival is in the range of 4-18 months. Current therapies include surgery, radiation therapy, chemotherapy, and multimodality therapy, but have yielded disappointing results [5]. It is hoped that a better understanding of MM biology may provide the rationale for new therapeutic strategies. In this regard, the development of tumor cell lines has been an important tool to study the biological properties of many tumors. However, only few mesothelioma cell lines have been established [6-10]. There is no specific marker for mesothelioma and antibodies that recognize molecules expressed by mesothelial cells and mesothelioma have limited specificity.

A cancer stem cell population in malignant tumors plays an essential role in tumor initiation, growth and recurrence [11,12]. It was demonstrated that cancer stem cells, capable of self-renewal and multilineage differentiation, are present in blood and solid tumors [13-15]. This clonogenic tumoral subpopulation is the only one able to originate a tumor mass containing the variety of differentiated cells present within the original tumor and, for this reason, these cells were described as cancer stem cells. This term may cause confusion, suggesting that the cell of origin is a normal stem cell, a question still unresolved. For this reason we prefer the term "tumor-initiating cells" or TICs, as opposed to the non-tumorigenic cancer cells.

Until now the presence of TICs in MM tumors has not been demonstrated. Moreover all the primary cell lines isolated so far were grown in medium containing at least 10% serum, and it was demonstrated that primary cells cultured under these high serum conditions lose their self-renewing properties, have no ability to differentiate, exhibit gene expression profiles that are different from tissue specific stem cells or the primary tumor they were derived from, and in some cases are neither clonogenic nor tumorigenic [16].

To obtain a better MM in vitro and in vivo model, we established MM cultures from fresh human pleural mesothelioma surgical specimens. The primary cell lines were expanded in vitro in a low serum proliferation-permissive medium and the expression of different markers as well as the tumorigenicity in immunodeficient mice was evaluated.

## Methods

### Patients and MM TICs culture

MM specimens were obtained from the Pneumology, Spedali Civili (Brescia, Italy) and the Villa Scassi Hospital (Genova Sampieradarena, Italy). Informed consent was obtained for all patients as approved by the Ethics Board. Pathologists diagnosed malignant Mesothelioma. Of the 35-mesothelioma samples collected 17 grew in vitro and only 4 in vivo. Two patients included in this study were at an advanced stage of MM, requiring only biopsy for the diagnosis. From two patients was obtained a portion of the resected malignant pleura sufficiently large to be able to undergo both in vitro cell culture and a portion was fixed in 10% buffered formalin and processed for the subsequent histological analysis. Tumor samples were obtained in sterile conditions avoiding apparently necrotic and fibrotic areas. The 4 patients, all male, underwent surgery or biopsy for the first time and never received chemotherapy or radiotherapy; all the sample were diagnosed as epithelioid MM. Tumor samples were immediately processed by mechanical disgregation and plated on gelatin coated flasks (Sigma, Saint Louis, MO, USA) in MCDB (Sigma), 2 mM L-glutamine (Gibco Ltd, Paisley, Scotland), recombinant human bFGF (10 ng/ml, Peprotech, London, UK), recombinant human EGF (20 ng/ml, Peprotech), 15 μg/ml insulin, 2 μg/ml heparin and 2% FBS (Euroclone, Devon UK). For subculture, 0.25% trypsin-EDTA (Euroclone, Devon UK) was used. If stromal cell growth was noted during initial growth, differential trypsinization was used to obtain a pure tumor cell population. The method is based on the treatment of the culture with trypsin for around 1 minute, with continuous microscopic observation. When the fibroblasts detach but the epithelial cells are still adherent the enzyme is inactivated with FCS containing medium. Pure populations of MM cells were obtained after three to four passages. All cell cultures showed the monolayer growth pattern, and population doubling times ranged from 63 to 68 hours. Cultured cells were frozen in 10% dimethylsulfoxide in fetal calf serum and stored in liquid nitrogen. The four cell cultures were used between the eighth and twelfth passage in culture.

### Immunofluorescence

Cells of all patients were plated on gelatin coated glass coverslips and then fixed with 4% paraformaldehyde, treated with PBS 10% NGS 0.5% triton X100 and then stained with the anti-SOX2 antibody (Chemicon, Temecula, CA, USA) followed by goat anti-mouse IgG (Alexa 488, Molecular Probes, Oslo, Norway) or rhodamine-conjugated goat anti-rabbit IgG (Jackson ImmunoResearch, West Grove, PA, USA). The cells were counterstained with Hoechst 33342 dye (Sigma) to identify nuclei.

Tumors cryosections were fixed in 4% PFA. Antibody staining was carried out following heat induced antigen retrieval using citrate buffer (pH 6.1) and with permeabilization in PBS containing 0.1% Triton X-100 and blocked in 10% normal Goat serum-PBS. After incubation with anti-Mesothelin antibody (Zymed Laboratories, San Francisco, CA, USA), sections were stained with the appropriate secondary antibodies and counterstained with Hoechst 33342 dye (Sigma) to identify all nuclei.

### MM xenografts and serial transplantation

10^3^-10^6 ^MM primary cells were intraperitoneally (i.p.) injected in 4-6 weeks old NOD-SCID mice (Charles River, Milan, Italy) to test their ability to grow in vivo. After several weeks cell growth was detected by the development of obvious ascites. In-vivo grown cells were harvested by intraperitoneal washing. A portion of the above-recovered tumor cells were injected i.p. into secondary NOD/SCID mice. Serial transplantation was carried out every two months until the fifth passage, using four mice per group. Mice were monitored for disease symptoms and were sacrificed by CO_2 _asphyxiation when ascites caused respiratory distress or they became extremely weak. All experiments were performed in compliance with guidelines approved by the Ethical Committee for animal use in cancer research at Istituto Nazionale Ricerca Cancro - Genova.

### Immunohistochemical analysis

For xenograft tumor analysis, tumor masses grown in vivo were collected from sacrificed mice, cryopreserved and 10-μm cryostat (Leica, Wetzlar, Germany) sections were cut. For WT-1 (Dako Cytomation, Denmark) staining, tumors cryosections were fixed in 4% PFA, treated with 0.4% pepsin in 0.2 N HCl, then with 3% H_2_O_2_-PBS protect from light, permeabilized in PBS 10% NGS 0.5% Triton X-100, incubated with primary antibody and then stained with anti-mouse EnVision-HRP (Dako). Signal was revealed with AEC substrate (Sigma).

For Calretinin (Dako) staining, sections were fixed in 4% PFA, then antibody staining was carried out following heat induced antigen retrieval using 10 mM Tris-Cl 1 mM EDTA pH9 and treatment with 0.3% H_2_O_2_-PBS protect from light. Cryosections were permeabilized in PBS 10% NGS 0.5% Triton X-100, incubated with primary antibody and then with biotynilated affinity-purified goat anti-mouse (BIOSPA, Milano Italy). Expression was detected using streptavidin/horseradish peroxidase (Jackson Immunoresearch). Signal was revealed with Chromogen 3-amino-9-ethylcarbazole (AEC) substrate.

For D2-40 staining (Dako), cryosections were fixed in 4% PFA, then antibody staining was carried out following heat induced antigen retrieval using Target Retrival Solution S1700 (Dako) and treatment with 3% H_2_O_2_-PBS protect from light. Cryosections were permeabilized in PBS 10% NGS 0.3% Triton X-100, incubated with primary antibody and then stained with anti-mouse EnVision-HRP and counterstained with Harris's Ematossilin (Bio-Optica). Signal was revealed with AEC substrate. Mouse IgG was used as a negative control for all the tissues. Digital images were captured by a Nikon Eclipse 80 i microscope (Nikon Europe, Lijnden, The Netherlands).

### Flow cytofluorimetric analysis

For cytofluorimetric analysis cultured MM cells were stained with the appropriate amount of PE or FITC-conjugated mAbs or PE or FITC- conjugated isotype-specific control antibody. MM cells were stained with the following antibodies: CD31, CD68, CD141, CD56, CD63, CD86, CD30, CD11b, CD46, CD184, EGFR, CD24, CD90, CD55, CD47, CD99, CD140b (BD Bioscience, San Jose, CA). 10,000 labeled cells were acquired and analyzed using a FACScan flow cytometer running CellQuest software (Becton Dickinson & Co, Mountain View, CA).

### Real-Time PCR

Total RNA was isolated using RNeasy Mini Kit (Qiagen, Milan, Italy), retrotranscribed using iScriptTM cDNA Synthesis Kit (Bio-Rad, Milano, Italy) and amplified with iQ Syber Green SuperMix (Bio-Rad) by PCR (iCYCLER, Bio-Rad) using the following primers: Nanog (forward 5'gaactctccaacatcctgaacctc3', reverse 5'ccttctgcgtcacaccattgc 3'), OCT4 (forward 5'tgtctccgtcaccactctg3', reverse 5'ctttctgttcccaattccttcc3'), Sema3A (forward 5'ggcatataatcagactcacttgtacgc3', reverse 5'cttgcatatctgacctattctagcgtg3'), NCAM2 (forward 5'agtggctccagtggcaaa3', reverse 5'ggctcccatcttcgtgatta3'), PDGFd (forward 5'aatgatgatgccaagcgttac3', reverse 5'gacctccagttgacagttcc3'), ABCA6 (forward 5'ggcaggcaatccaggcagtc3', reverse 5'acacggtcacacaaggcttcc3'), PDGFRb (forward 5'gtcctgcctgtccttctac3', reverse 5'tgtccttgctgctgatgg3'), DKK1 (forward 5'agtccttctgagatgatgg3', reverse 5'ggttcttgatagcgttgg3'), Mesothelin (MSTN) (forward 5'ggcacagaagaatgtcaag3', reverse 5'atagcagcaggtccaatg3'), calretinin (CALB2) (5'aacttccttctgtgcttc3', reverse 5'tatggtttgggtgtattcc3'). Samples were normalized using L41 primers (forward 5'agtggaggaagaagcgaatg3', reverse 5'tttatgagcaaggtgggtctc3'). The four primary cell cultures were analyzed together with positive and negative controls. Data presented here are means of biological and technical duplicates.

### Cytogenetic analysis

Metaphases for cytogenetic analysis were prepared from the first xenograft of the MM cell cultures. Chromosome preparations were obtained by standard cytogenetic techniques. For Q-banding metaphase spreads were stained by 4',6-diamidino-2-phenylindole (DAPI) (Sigma-Aldrich) and counterstained with actinomycin D (Sigma-Aldrich) [17] (Scheizer 1994). and karyotyped according to the International System for Human Cytogenetic Nomenclature [18] (Shaffer LG, 2005) using the Cytovision Karyotyping software (Applied Imaging, Newcastle-upon-Tyne, UK).

## Results

### Establishment of human MM cell cultures

**Figure 1:**
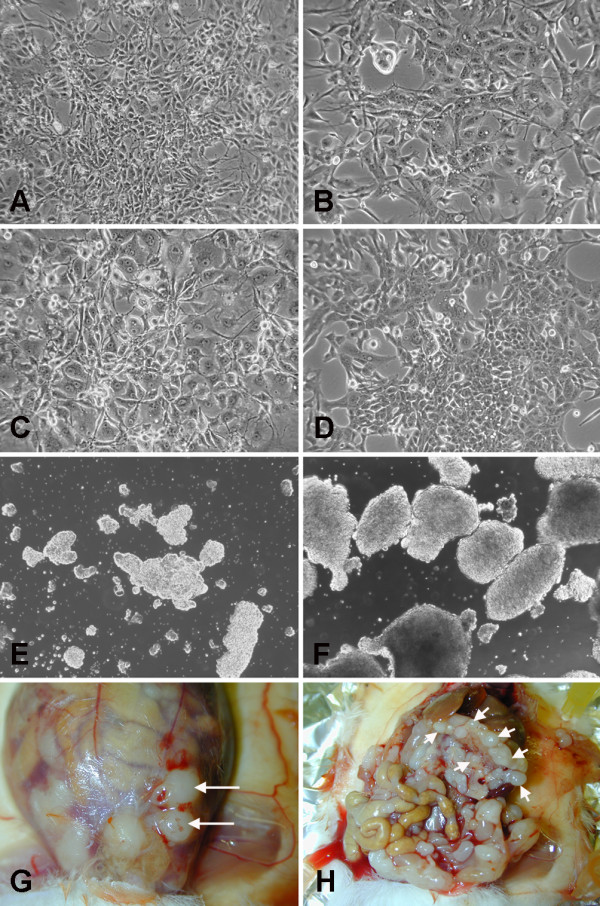
**In vitro and in vivo behavior of MM cells**. A-D: Morphological appearance of the MM1, MM2, MM3 and MM4 cell cultures respectively. E, F: free floating aggregates recovered from primary xenografts. Solid tumors grown in the abdominal wall at the site of injection (G), or in the retro gastric space (H).

Seventeen of 35 original tumors could be cultured under low-serum conditions, the remaining failing to grow probably because of the reduced number of collected cells. The new MM cell cultures were established from 17 patients and have been maintained in culture for up to 36 months. Plated cells exhibit polygonal and epithelial-like shape under phase-contrast microscope (Fig. 1A-D). They were successfully cultured in a low serum proliferation-permissive medium (see Materials and Methods) as spheres and as adherent monolayers on gelatin-coated flasks. After thawing, the cryopreserved cells were able to propagate in culture without noticeable change in growth and morphology. The doubling time was evaluated at about 65 h. In all subsequent experiments the four cell cultures were used between the fifth and twelfth passage in culture.

### *In vivo *growth of human MM cell cultures

To assess the tumorigenic properties of the MM cultures, 10^3^-10^6 ^MM primary cells were intraperitoneally injected, within the fifth passage, in immunodeficient NOD/SCID mice. Of the 17 established cell cultures only four were able to grow in vivo, and were named MM1 to MM4. After 3 to 6 months (Table 1), all cultured cell tumors were generated with histological properties resembling the primitive neoplasia. Animals that received mesothelioma cells by i.p. injection developed moderate ascites; the vast majority of cells grew as free-floating aggregates (Fig. 1E, F) and solid tumors formed in the abdominal wall at the site of injection, recapitulating the described ability of invasion through the needle biopsy tract, typical features of malignant mesothelioma [19,20], or in the retro gastric space; peritoneal metastasis were seldom observed (Fig. 1G, H). The mean survival time was 120 days for mice injected with MM1 cells, 150 days for mice injected with the MM2 cells, 210 days for mice injected with the MM3 cells and 110 days for mice injected with MM4 cells. Survival time was related to the number of injected cells, as shown by the results obtained injecting different numbers of MM1 cells, ranging from 10^3 ^to 10^6^. For the lowest number of injected cells, although the tumor take rate remained 100%, the survival time extended from 108 to 166 days. Cells isolated from dissociated xenograft tumors and re-cultured under stem cells conditions were able to form spheres and, when injected back into new recipient mice, not only retained the ability to generate new tumors, but also showed an increased tumorigenic potential, as shown by the shorter time required for tumor development (Tab. 1).

**Table 1:** Survival time of mice serially transplanted.

	1^st ^transplant	2^nd^ transplant	3^rd ^transplant	4^th^ transplant
**MM1**	180 ± 20	130 ± 6	117 ± 3	76 ± 8

**MM2**	150 ± 16	122 ± 8	80 ± 5	70 ± 12

**MM3**	210 ± 28	105 ± 11	150 ± 15	41 ± 6

**MM4**	84 ± 8	92 ± 5		

The four cell cultures were intraperitoneally injected into NOD/SCID mice (four mice per group). Data show the mean survival time indicated as days.

### Immunohistochemical analysis

**Figure 2:**
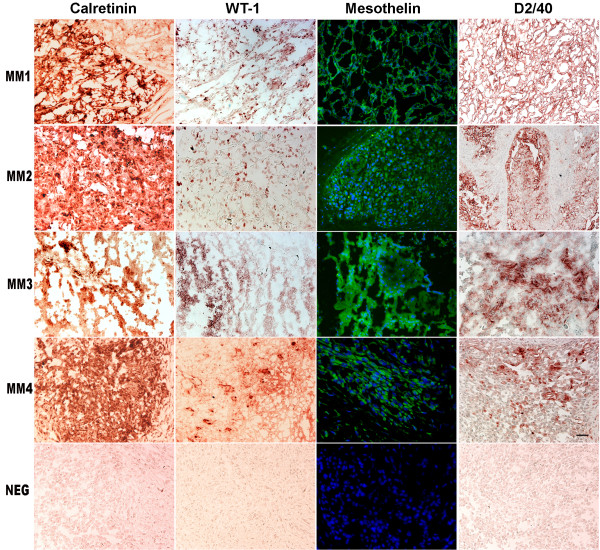
**MMs immunohistology**. Solid tumors recovered from primary xenografts of the four MMs cell cultures stained with the indicated antibodies. Negative controls for MM4 are included in the bottom row. Scale bar 100 μm.

MM cells were injected into the peritoneal cavity of NOD/SCID mice and tumor samples were collected for immunohistochemical analysis. To confirm their mesothelial origin, a panel of four markers typical of mesothelioma was used: mesothelin, calretinin, D2-40 and WT-1. Xenografts were strongly positive for calretinin and moderately positive for D2-40, mesothelin and WT-1 (Fig. 2). This immunohistochemical profile is diagnostic for human malignant mesothelioma. Cells isolated from dissociated xenograft tumors and re-cultured in a low serum proliferative-permissive medium, are weakly positive for the same markers (data not shown).

### MM cells expression analysis

**Figure 3:**
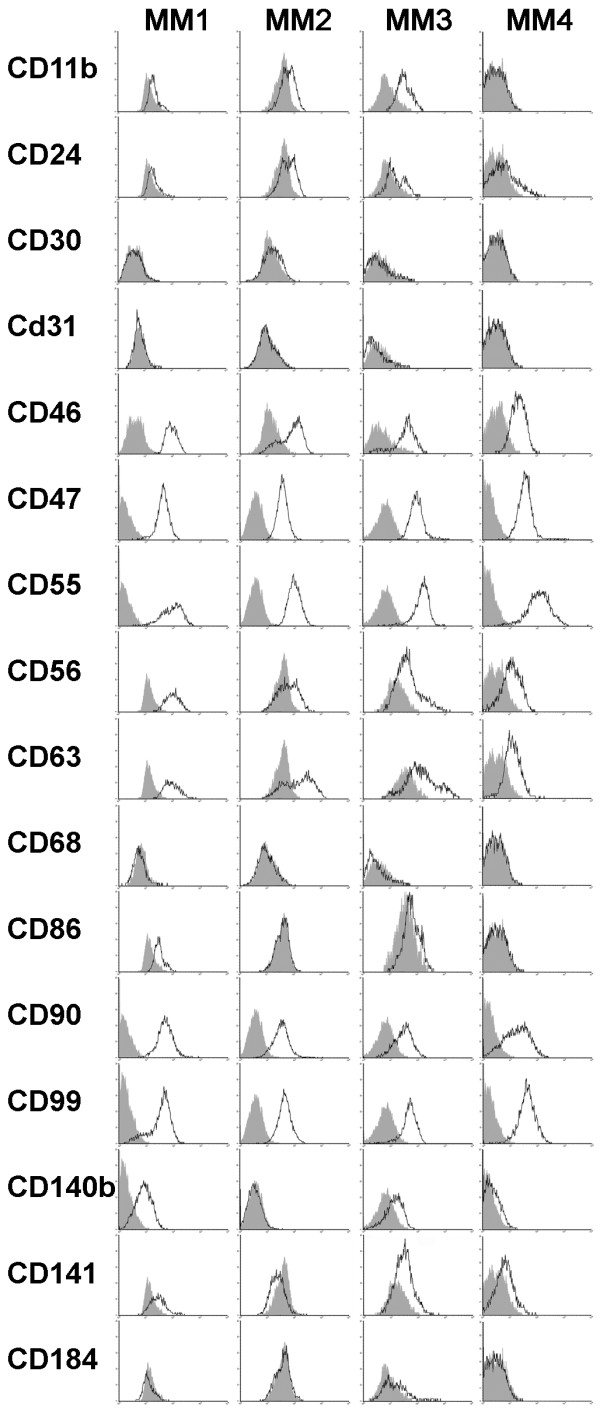
**In vitro and in vivo behavior of MM cells**. MM cells recovered from primary xenografts were analyzed by FACS for the expression of the indicated molecules using the appropriate amount of PE or FITC-conjugated mAbs. Grey profiles refer to cells incubated with PE or FITC- conjugated isotype-specific control antibody.

**Figure 4:**
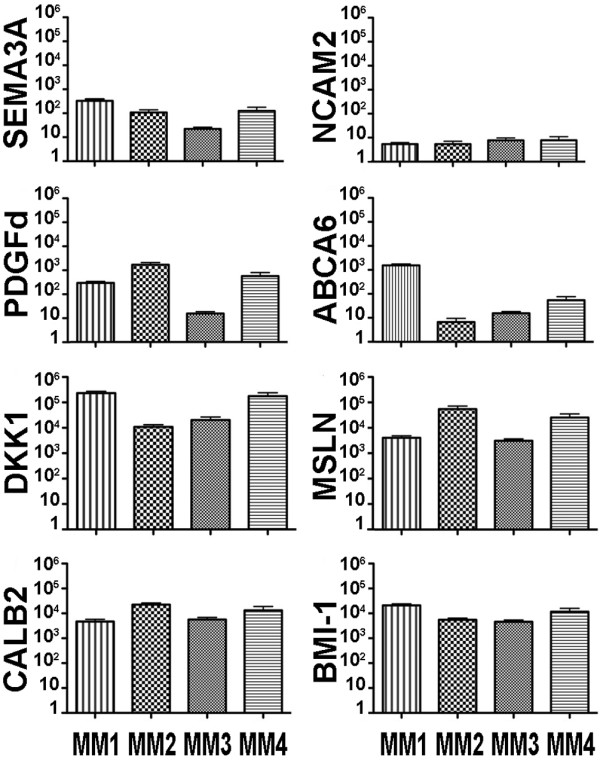
**MMs Real Time PCR**. Expression of indicated genes analyzed by qRT-PCR. Data are shown as ratio between the indicated gene and an housekeeping gene (L41). Bars indicate the Standard Deviation, values on Y axis are in log-scale.

Cells recovered from the primary xenografts and cultured for 3 to 5 passages, to deplete the possible contaminating mouse macrophages, were analyzed by FACS for the expression of surface markers already known to be expressed by mesothelioma cell lines or by other kinds of neoplasias. All cell cultures were more than 90% positive for CD47, CD55 and CD99, between 65 and 95% for CD90 and CD46, between 30 and 92% for CD56 and CD63, between 5 and 60% for CD140b, between 0 and 20% for CD141. The cells were instead negative or dim for CD11b, CD24, CD30, CD31, CD68, CD86 and CD184 (CXCR4), (Fig. 3). Further gene expression studies were performed by real time PCR on the four MMs, in order to evaluate the cell type-specific expression of various markers. MM cell cultures expressed high mRNA levels of the mesothelioma markers mesothelin (MSLN), calretinin (CALB2), the WNT1 antagonist DKK1 and the stem cell marker BMI-1. The four cell cultures also express PDGFd, SEMA3A and ABCA6 at intermediate levels and NCAM2 at low levels (Fig. 4) but were negative for other stem cell markers such as SOX2, Nanog, Oct4 and CD133, (data not shown).

### Cytogenetic analysis

The karyotypes of the four cultures are reported in table 2. The karyotypes are hypodiploid (MM 4), hypotetraploid (MM2 and MM3) or mixed hypodiploid and hypotetraploid (MM1). All the karyotypes appeared very rearranged. In most of the cases, the short arm of chromosome 3 and the short arm of chromosome 1 are deleted or involved in rearrangements. In three cases out of four the long arm of chromosome 6 is rearranged as well as the long arm of chromosome 12. As far as chromosome losses, in all cases chromosome 8, 14 and 15 are lost. The chromosome rearrangements distribution in the individual cultures demonstrate an oligoclonal composition.

**Table 2:** Q-banding karyotypes of the four MM cell cultures recovered form primary transplants.

MM1	39-40, XY dic(1;4)(p12;q31), t(1;19)(p13;p13.1),2, del(2)(p21), del(3)(p14), del(3)(q13), del(6)(q14q22),-8, del(10)(q2425), der(11)t(11;18)(q23;q11.2), der(12)t(12;?)(q22;?), -13,-13,-14,-15,-17,-18,-18,+4-5 m [3cp]; 76-77, XXYY idemx2 [2cp]
MM2	71-102, XXY der(1)dup(p13p32)inv(q12p33)x2,-2, t(3;?)(p0;?), i(3q),-4,+5,-8, del(9)(p21),-9,11, der(12) del(12)(p13)t(12;?)(q24;?)x2, der(13)t(13;?)(p0;?),-13,-14,-15,-16,-17,-18,-19-20,-21,+10 m [4cp]

MM3	58-78, XX-Y, i(1)(q0),-1, der(3)t(3;12)(q0;p0), del(4)(q32),-4,-5, del(6)(q16q21), -6,-7,-8,-9, del(10)(q24q26),+11,-12,-13,-14,-15,-16,-18,-22,+5-10 m [5cp]

MM4	36-37, XY del(1)(p32), t(1;11)(p32;q22)dup(11)(q14q22), del(3)(p21), t(3;22)(q11.2;q13),-4,-5, add(6) (q27),-8,-9,-9,+der(12)dup(12)(q21),-14,-14,-15,-15,-16,-17,17, i(18q), der(19)t(19;?)(q13.2;?), add(21)(p12),+1-6 m [3cp]

## Discussion

The development of tumor cell lines has been an important tool in establishing suitable in vitro models for studying the biological properties of many tumors. All mesothelioma cell lines derived so far were established in-high serum growth media. Tumor cells expanded under such conditions, however, often display phenotypic characteristics and genetic aberrations due to the long term in vitro culture in the presence of calf serum and are very different to those found within the corresponding primary human tumor, also in their gene expression profile.

We have derived and characterized four human mesothelioma cell cultures that retain tumor initiating cell properties, using the chemically defined medium MCDB201 supplemented with 2% FCS, EGF and FGF. A low serum medium and selected mitogens were used for their known ability to encourage self-renewal by preventing in vitro commitment to differentiation, and to preserve the ability to form tumors in immunodeficient mice that recapitulate the heterogeneity normally seen on human cancers [16,21].

The growth and morphological properties of these new established mesothelioma cell cultures are heterogeneous. Some of them grow as fairly firmly attached monolayers whereas others grow as both floating and loosely attached cultures.

The MM1-4 cell cultures described here are able to engraft after pseudo orthotopic intraperitoneal transplantation in immunodeficient mouse, where they mainly grow as free floating cells that eventually aggregate, recapitulating a characteristic that is thought to be typical of normal mesothelial stem/progenitor cells [22]. When as low as 1000 cells were injected the tumor take rate was not affected, indicating that our cell cultures are highly enriched in tumor initiating cells when compared to the other available MM cell lines, which require at least 10^6 ^cells to obtain the same take rate [23]. Their ability to engraft after serial transplantation is a hallmark of tumor initiating cells. The faster development of tumor after serial transplantation may be due to a progressive increase of the TICs fraction or to an in vivo selection of the more aggressive cells. To avoid this selection of fast proliferating cells and to completely deplete the contaminating fibroblasts present in primary cultures, all the experiments were performed with cells recovered from the first xenograft. To determine whether MM cell cultures display genomic alterations characteristic of mesothelioma, we performed cytogenetic analyses of the cell cultures recovered from primary xenografts. The results confirmed their human origin and showed many and varied structural and numeric abnormalities. The disease develops for decades, during which the neoplastic cells accumulate a variety of chromosomal aberrations. Cytogenetic analysis revealed rearrangement of the short arm of chromosome 1, an aberration frequently reported in mesothelioma karyotypes (56% of the cases), as well as rearrangements of the short arm of chromosome 3 that are found in 51% of the cases, deletion accounting for nearly half of them (48%)[24]. Sandberg and Bridge [25] proposed a scheme for the stepwise process of genetic changes leading to the full development of malignant mesothelioma. In this scheme a deletion in the short arm of chromosome 3 is a late event and deletion of chromosome 1 is mainly found in metastatic tumors.

In order to better characterize the MM cell cultures we performed a phenotypic characterization by FACS analysis using antibodies against membrane proteins. Among the surface markers known to be expressed by mesothelioma cell lines, our cell cultures were strongly positive for CD46 [26], CD47 [27], CD56 [28] and CD63 [29], while showed a variable positivity for CD140b [30] and CD141 [31]. The four MM cell cultures were also strongly positive for some markers never described before in mesothelioma cell lines, including CD55, CD90 and CD99 that may represent new markers potentially useful for the isolation of cancer stem cells from MM. CD55 together with CD46 are complement regulatory proteins. Cells that express complement regulatory proteins are able to modulate their sensitivity to complement-mediated lysis and their ability to survive into a proinflammatory environment in which C3 activation frequently occurs. It has been demonstrated that a reduction in CD46 results in increased C3b deposition on apoptotic cells, which are then killed by macrophages. The complement-regulatory proteins are also able to regulate T cell function inhibiting the immune response against cancer. CD55 was also found expressed on side-population (SP) cells of mammary carcinoma cell lines and used for isolation of cancer stem cells. Cells expressing high level of CD55 were resistant to apoptosis induced by serum depletion as in the case of SP cells. The identification of CD90 on our cells, a marker known to be expressed on subsets of hematopoietic and non-hematopoietic stem cells, raises the possibility that CD90 expression identifies a highly proliferative and/or stem cell-like component of these neoplasm. CD90 is also expressed on mesenchymal stem cells and fibroblasts not committed to adipogenesis.

Immunoreactivity of CD99 was detected in invasive malignant melanoma, in pancreatic endocrine tumors, Ewing's sarcoma and gastric cancer. A splice variant of CD99 increases motility and MMP-9 expression of human breast cancer cells, in osteosarcoma and prostate cancer. In osteosarcoma and prostate cancer, cells overexpressing the long CD99wt isoform inhibited and delayed formation of lung or bone metastases. The high level of CD99 on MM cells may explain the absence of long distance metastasis.

CD140b (PDGFRβ) is of special interest since the a subunit of the receptor is predominantly expressed in benign mesothelial cells, whereas expression of the β subunit is found in MM cell lines and clinical specimens [32]. An inverse correlation between the expression of PDGFRb and PDGFd was observed on our MM cells. This observation suggests that cells expressing low levels of receptor needs to produce high levels of ligand, supporting the importance of this autocrine pathway in MM, a possible therapeutic target.

By real time PCR we found that our cell cultures expressed high mRNA levels of typical mesothelioma markers as mesothelin (MSLN) and calretinin (CALB2), of BMI-1, a stemness marker and of DKK1, a potent Wingless [WNT] inhibitor. It is interesting to note that, in a previous report [33], DKK1 was used to induce apoptosis of MM cells by antagonizing Wnt signaling independently of β-catenin. Our cell cultures express DKK1 at high levels, like other tumors such as Wilms' tumor, osteosarcomas, hepatocellular carcinomas and prostate cancer [34-36]. It is already known that Wnt signaling is an osteoinductive signal that promotes differentiation of osteo-progenitors and that, in human mesenchymal stem cell the inhibition of Wnt signaling with recombinant soluble DKK1 induces spontaneous transformation and tumorigenicity [37]. Other reports indicates that sustained Wnt signaling inhibits neural differentiation while inducing differentiation along meso/endoderm lineages in human and mouse embryonic stem cells [38]. It is therefore conceivable to hypothesize that mesothelium belongs to the developmental lineage group of cells that are induced to differentiate by Wnt signaling. Thus, the inhibitory effect of DKK1 would maintain stemness and inappropriate persistent DKK1 and BMI-1 expression would ultimately lead to malignant transformation, by keeping the cells in an undifferentiated, "stem-like" cellular state.

## Conclusions

In conclusion, this paper describes the establishment and characterization of four new mesothelioma cell cultures derived from male patients that never received chemotherapy or radiotherapy. Our cells, cultured under stemness conditions, are able to survive and grow in low serum conditions, although at a slower rate than that observed under high serum conditions. When transplanted into immunodeficient mice these cells are able to engraft and form tumors that are phenocopies of the original tumor. The cells expressed mesothelioma markers together with some typical genes involved in stemness such as BMI-1 and CD90. The expression of these stem cell markers may reflect the presence in these cultures of a higher proportion of TICs compared to the mesothelioma cell lines available to date which have been kept in high serum media and passaged for long periods. This higher "stemness" is supported by the fact that 10^3 ^cells are able to achieve 100% tumor take, a thousand-fold less than required by the other available MM cell lines. For these reasons, these MM cell cultures may provide a valuable in vitro and in vivo model to study the biology of this poorly understood deadly tumor.

## Competing interests

The authors declare that they have no competing interests.

## Authors' contributions

AM, AD, LM and GC contributed to the conception and design of the study, analysis and interpretation of the results, drafting of the final manuscript. AM and AD performed the in vitro and in vivo studies. DM carried out the immunohistochemistry studies. AZ performed the cytogenetic analysis. All authors have read and approved the final manuscript.

## Pre-publication history

The pre-publication history for this paper can be accessed here:

http://www.biomedcentral.com/1471-2407/10/54/prepub

## References

[B1] Simsir A Fetsch P Bedrossian CW Ioffe OB Abati A Absence of SV-40 large T antigen (Tag) in malignant mesothelioma effusions: an immunocytochemical study Diagn Cytopathol 2001 25 4 203 207 10.1002/dc.2039 11599101

[B2] Bocchetta M Di Resta I Powers A Fresco R Tosolini A Testa JR Pass HI Rizzo P Carbone M Human mesothelial cells are unusually susceptible to simian virus 40-mediated transformation and asbestos cocarcinogenicity Proc Natl Acad Sci USA 2000 97 18 10214 10219 10.1073/pnas.170207097 10954737 PMC27818

[B3] Cristaudo A Foddis R Vivaldi A Buselli R Gattini V Guglielmi G Cosentino F Ottenga F Ciancia E Libener R SV40 enhances the risk of malignant mesothelioma among people exposed to asbestos: a molecular epidemiologic case-control study Cancer Res 2005 65 8 3049 3052 15833832 10.1158/0008-5472.CAN-04-2219

[B4] Ismail-Khan R Robinson LA Williams CC Jr Garrett CR Bepler G Simon GR Malignant pleural mesothelioma: a comprehensive review Cancer Control 2006 13 4 255 263 17075562 10.1177/107327480601300402

[B5] West SD Lee YC Management of malignant pleural mesothelioma Clin Chest Med 2006 27 2 335 354 10.1016/j.ccm.2006.01.004 16716822

[B6] Manning LS Whitaker D Murch AR Garlepp MJ Davis MR Musk AW Robinson BW Establishment and characterization of five human malignant mesothelioma cell lines derived from pleural effusions Int J Cancer 1991 47 2 285 290 10.1002/ijc.2910470219 1703129

[B7] Orengo AM Spoletini L Procopio A Favoni RE De Cupis A Ardizzoni A Castagneto B Ribotta M Betta PG Ferrini S Establishment of four new mesothelioma cell lines: characterization by ultrastructural and immunophenotypic analysis Eur Respir J 1999 13 3 527 534 10.1183/09031936.99.13352799 10232421

[B8] Pass HI Stevens EJ Oie H Tsokos MG Abati AD Fetsch PA Mew DJ Pogrebniak HW Matthews WJ Characteristics of nine newly derived mesothelioma cell lines Ann Thorac Surg 1995 59 4 835 844 10.1016/0003-4975(95)00045-M 7695406

[B9] Usami N Fukui T Kondo M Taniguchi T Yokoyama T Mori S Yokoi K Horio Y Shimokata K Sekido Y Establishment and characterization of four malignant pleural mesothelioma cell lines from Japanese patients Cancer Sci 2006 97 5 387 394 10.1111/j.1349-7006.2006.00184.x 16630136 PMC11158456

[B10] Zeng L Fleury-Feith J Monnet I Boutin C Bignon J Jaurand MC Immunocytochemical characterization of cell lines from human malignant mesothelioma: characterization of human mesothelioma cell lines by immunocytochemistry with a panel of monoclonal antibodies Hum Pathol 1994 25 3 227 234 10.1016/0046-8177(94)90192-9 7512071

[B11] Dalerba P Cho RW Clarke MF Cancer stem cells: models and concepts Annu Rev Med 2007 58 267 284 10.1146/annurev.med.58.062105.204854 17002552

[B12] Cho RW Clarke MF Recent advances in cancer stem cells Curr Opin Genet Dev 2008 18 1 48 53 10.1016/j.gde.2008.01.017 18356041

[B13] Al-Hajj M Wicha MS Benito-Hernandez A Morrison SJ Clarke MF Prospective identification of tumorigenic breast cancer cells Proc Natl Acad Sci USA 2003 100 7 3983 3988 10.1073/pnas.0530291100 12629218 PMC153034

[B14] Bonnet D Dick JE Human acute myeloid leukemia is organized as a hierarchy that originates from a primitive hematopoietic cell Nat Med 1997 3 7 730 737 10.1038/nm0797-730 9212098

[B15] Singh SK Clarke ID Hide T Dirks PB Cancer stem cells in nervous system tumors Oncogene 2004 23 43 7267 7273 10.1038/sj.onc.1207946 15378086

[B16] Lee J Kotliarova S Kotliarov Y Li A Su Q Donin NM Pastorino S Purow BW Christopher N Zhang W Tumor stem cells derived from glioblastomas cultured in bFGF and EGF more closely mirror the phenotype and genotype of primary tumors than do serum-cultured cell lines Cancer Cell 2006 9 5 391 403 10.1016/j.ccr.2006.03.030 16697959

[B17] Schweizer D Ambros PF Chromosome banding. Stain combinations for specific regions Methods Mol Biol 1994 29 97 112 7518289 10.1385/0-89603-289-2:97

[B18] Shaffer LGTN ISCN 2005: An International System for Human Cytogenetic Nomenclature 2005 Basel, Switzerland: Karger

[B19] Chahinian AP Pajak TF Holland JF Norton L Ambinder RM Mandel EM Diffuse malignant mesothelioma. Prospective evaluation of 69 patients Ann Intern Med 1982 96 6 Pt 1 746 755 7091938 10.7326/0003-4819-96-6-746

[B20] Hillerdal G Malignant mesothelioma 1982: review of 4710 published cases Br J Dis Chest 1983 77 4 321 343 10.1016/0007-0971(83)90068-2 6357260

[B21] Griffero F Daga A Marubbi D Capra MC Melotti A Pattarozzi A Gatti M Bajetto A Porcile C Barbieri F Different response of human glioma tumor-initiating cells to epidermal growth factor receptor kinase inhibitors J Biol Chem 2009 284 11 7138 7148 10.1074/jbc.M807111200 19147502 PMC2652334

[B22] Herrick SE Mutsaers SE Mesothelial progenitor cells and their potential in tissue engineering Int J Biochem Cell Biol 2004 36 4 621 642 10.1016/j.biocel.2003.11.002 15010328

[B23] Bertino P Piccardi F Porta C Favoni R Cilli M Mutti L Gaudino G Imatinib mesylate enhances therapeutic effects of gemcitabine in human malignant mesothelioma xenografts Clin Cancer Res 2008 14 2 541 548 10.1158/1078-0432.CCR-07-1388 18223230

[B24] Mitelman Database of Chromosome Aberrations in Cancer (2009) Mitelman F, Johansson B, Mertens F http://cgap.nci.nih.gov/Chromosomes/Mitelman Accessed on 2009

[B25] Sandberg AA Bridge JA Updates on the cytogenetics and molecular genetics of bone and soft tissue tumors. Mesothelioma Cancer Genet Cytogenet 2001 127 2 93 110 10.1016/S0165-4608(01)00432-0 11425448

[B26] Gauvrit A Brandler S Sapede-Peroz C Boisgerault N Tangy F Gregoire M Measles virus induces oncolysis of mesothelioma cells and allows dendritic cells to cross-prime tumor-specific CD8 response Cancer Res 2008 68 12 4882 4892 10.1158/0008-5472.CAN-07-6265 18559536

[B27] Gordon GJ Rockwell GN Jensen RV Rheinwald JG Glickman JN Aronson JP Pottorf BJ Nitz MD Richards WG Sugarbaker DJ Identification of novel candidate oncogenes and tumor suppressors in malignant pleural mesothelioma using large-scale transcriptional profiling Am J Pathol 2005 166 6 1827 1840 15920167 10.1016/S0002-9440(10)62492-3 PMC1363736

[B28] Lantuejoul S Laverriere MH Sturm N Moro D Frey G Brambilla C Brambilla E NCAM (neural cell adhesion molecules) expression in malignant mesotheliomas Hum Pathol 2000 31 4 415 421 10.1053/hp.2000.6552 10821486

[B29] Hegmans JP Bard MP Hemmes A Luider TM Kleijmeer MJ Prins JB Zitvogel L Burgers SA Hoogsteden HC Lambrecht BN Proteomic analysis of exosomes secreted by human mesothelioma cells Am J Pathol 2004 164 5 1807 1815 15111327 10.1016/S0002-9440(10)63739-X PMC1615654

[B30] Kothmaier H Quehenberger F Halbwedl I Morbini P Demirag F Zeren H Comin CE Murer B Cagle PT Attanoos R EGFR and PDGFR differentially promote growth in malignant epithelioid mesothelioma of short and long term survivors Thorax 2008 63 4 345 351 10.1136/thx.2007.085241 18086752

[B31] Ordonez NG Immunohistochemical diagnosis of epithelioid mesothelioma: an update Arch Pathol Lab Med 2005 129 11 1407 1414 16253021 10.5858/2005-129-1407-IDOEMA

[B32] Versnel MA Claesson-Welsh L Hammacher A Bouts MJ Kwast TH van der Eriksson A Willemsen R Weima SM Hoogsteden HC Hagemeijer A Human malignant mesothelioma cell lines express PDGF beta-receptors whereas cultured normal mesothelial cells express predominantly PDGF alpha-receptors Oncogene 1991 6 11 2005 2011 1658707

[B33] Lee AY He B You L Xu Z Mazieres J Reguart N Mikami I Batra S Jablons DM Dickkopf-1 antagonizes Wnt signaling independent of beta-catenin in human mesothelioma Biochem Biophys Res Commun 2004 323 4 1246 1250 10.1016/j.bbrc.2004.09.001 15451431

[B34] Hall CL Daignault SD Shah RB Pienta KJ Keller ET Dickkopf-1 expression increases early in prostate cancer development and decreases during progression from primary tumor to metastasis Prostate 2008 68 13 1396 1404 10.1002/pros.20805 18561248 PMC3260942

[B35] Patil MA Chua MS Pan KH Lin R Lih CJ Cheung ST Ho C Li R Fan ST Cohen SN An integrated data analysis approach to characterize genes highly expressed in hepatocellular carcinoma Oncogene 2005 24 23 3737 3747 10.1038/sj.onc.1208479 15735714

[B36] Wirths O Waha A Weggen S Schirmacher P Kuhne T Goodyer CG Albrecht S Von Schweinitz D Pietsch T Overexpression of human Dickkopf-1, an antagonist of wingless/WNT signaling, in human hepatoblastomas and Wilms' tumors Lab Invest 2003 83 3 429 434 12649343 10.1097/01.lab.0000059926.66359.bd

[B37] Matushansky I Hernando E Socci ND Mills JE Matos TA Edgar MA Singer S Maki RG Cordon-Cardo C Derivation of sarcomas from mesenchymal stem cells via inactivation of the Wnt pathway J Clin Invest 2007 117 11 3248 3257 10.1172/JCI31377 17948129 PMC2030456

[B38] Bakre MM Hoi A Mong JC Koh YY Wong KY Stanton LW Generation of multipotential mesendodermal progenitors from mouse embryonic stem cells via sustained Wnt pathway activation J Biol Chem 2007 282 43 31703 31712 10.1074/jbc.M704287200 17711862

